# Physiological and Biochemical Effects of Intrinsically High and Low Exercise Capacities Through Multiomics Approaches

**DOI:** 10.3389/fphys.2019.01201

**Published:** 2019-09-18

**Authors:** Yu-Tang Tung, Yi-Ju Hsu, Chen-Chung Liao, Shang-Tse Ho, Chi-Chang Huang, Wen-Ching Huang

**Affiliations:** ^1^Graduate Institute of Metabolism and Obesity Sciences, Taipei Medical University, Taipei, Taiwan; ^2^Nutrition Research Center, Taipei Medical University Hospital, Taipei, Taiwan; ^3^Cell Physiology and Molecular Image Research Center, Wan Fang Hospital, Taipei Medical University, Taipei, Taiwan; ^4^Graduate Institute of Sports Science, National Taiwan Sport University, Taoyuan City, Taiwan; ^5^Proteomics Research Center, National Yang-Ming University, Taipei, Taiwan; ^6^Agricultural Biotechnology Research Center, Academia Sinica, Taipei, Taiwan; ^7^Department of Exercise and Health Science, National Taipei University of Nursing and Health Sciences, Taipei, Taiwan

**Keywords:** intrinsic exercise capacity, physical activities, gut microbiota, transcriptome, proteome

## Abstract

Regular exercise prevents lipid abnormalities and conditions such as diabetes mellitus, hypertension, and obesity; it considerably benefits sedentary individuals. However, individuals exhibit highly variable responses to exercise, probably due to genetic variations. Animal models are typically used to investigate the relationship of intrinsic exercise capacity with physiological, pathological, psychological, behavioral, and metabolic disorders. In the present study, we investigated differential physiological adaptations caused by intrinsic exercise capacity and explored the regulatory molecules or mechanisms through multiomics approaches. Outbred ICR mice (*n* = 100) performed an exhaustive swimming test and were ranked based on the exhaustive swimming time to distinguish intrinsically high- and low-capacity groups. Exercise performance, exercise fatigue indexes, glucose tolerance, and body compositions were assessed during the experimental processes. Furthermore, the gut microbiota, transcriptome, and proteome of soleus muscle with intrinsically high exercise capacity (HEC) and low exercise capacity (LEC) were further analyzed to reveal the most influential factors associated with differential exercise capacities. HEC mice outperformed LEC mice in physical activities (exhaustive swimming and forelimb grip strength tests) and exhibited higher glucose tolerance than LEC mice. Exercise-induced peripheral fatigue and the level of injury biomarkers (lactate, ammonia, creatine kinase, and aspartate aminotransferase) were also significantly lower in HEC mice than in LEC mice. Furthermore, the gut of the HEC mice contained significantly more *Butyricicoccus* than that of the LEC mice. In addition, transcriptome data of the soleus muscle revealed that the expression of microRNAs that are strongly associated with exercise performance-related physiological and metabolic functions (i.e., miR-383, miR-107, miR-30b, miR-669m, miR-191, miR-218, and miR-224) was higher in HEC mice than in LEC mice. The functional proteome data of soleus muscle indicated that the levels of key proteins related to muscle function and carbohydrate metabolism were also significantly higher in HEC mice than in LEC mice. Our study demonstrated that the mice with various intrinsic exercise capacities have different gut microbiome as well as transcriptome and proteome of soleus muscle by using multiomics approaches. The specific bacteria and regulatory factors, including miRNA and functional proteins, may be highly correlated with the adaptation of physiological functions and exercise capacity.

## Introduction

Unhealthy diet and lack of exercise cause more than 300,000 deaths per year. A sedentary lifestyle is considered an independent risk factor for cardiovascular disease (Blair et al., 1989; McGinnis and Foege, 1993). Regular physical activity can reduce both morbidity and all-cause mortality, including preventing dyslipidemia, diabetes, hypertension, and obesity development (Jennings et al., 1989; Blair et al., 1995). Individuals exhibit variable responses to exercise, which may be primarily mediated through genetic variations (Bouchard et al., 1999). Furthermore, genes determine intrinsic exercise capacity in an untrained state (Braith et al., 1994; Krushkal et al., 1999). High intrinsic exercise capacity is related to genes that determine adaptive responses to exercise (Braith et al., 1994; Bouchard et al., 2000). In addition, aerobic endurance is affected by genetic and environmental factors (Falconer and Mackay, 1996). Researchers have also demonstrated an increased prevalence of interrelated chronic metabolic diseases among individuals with low intrinsic exercise capacity, including insulin resistance, type 2 diabetes mellitus, obesity, and coronary heart disease (Schwarzer et al., 2010; Pekkala et al., 2017; Koch and Britton, 2018). Therefore, aerobic capacity may be closely related to multiple chronic diseases.

Endurance exercise includes running, cross-country skiing, cycling, swimming, and other aerobic exercises, and it can be defined as cardiovascular exercise (Joyner and Coyle, 2008). The physiological and biochemical requirements of endurance exercise elicit muscle- and system-based responses. The main adaptations caused by endurance exercise are improved mechanical, metabolic, neuromuscular, and contractile function in muscles; rebalanced electrolytes (Russell et al., 2013); reduced glycogen stores (Munoz et al., 2010); and increased mitochondrial biogenesis in muscle (Snow et al., 1981). In addition, endurance exercise may cause an increase in oxidative stress, intestinal permeability, muscle damage, systemic inflammation, and immune responses (Davies et al., 1981; Mach and Fuster-Botella, 2017).

The human intestine contains numerous microorganisms that significantly affect host nutrition, metabolic function, intestinal development, immune system function, and epithelial cell maturation (Hooper and Gordon, 2001). The gut microbiota promotes food digestion and absorption (Hsu et al., 2015), and the microbiota digests and subsequently ferments complex carbohydrates in the colon into short-chain fatty acids, such as *n-*butyrate, acetate, and propionate. A study found that elite male rugby players had lower levels of Bacteroidetes and higher levels of Firmicutes than healthy non-athletic individuals (Clarke et al., 2014). Regardless of diet, exercise increased the percentage of Bacteroides and reduced the percentage of Firmicutes (Evans et al., 2014). In ovariectomized female high-capacity runner (HCR) and low-capacity runner (LCR) breed rats, exercise intervention caused differential effects on host metabolism and gut microbial communities. The abundance of Firmicutes, Proteobacteria, and Cyanobacteria caused shifts in ovariectomized LCR, but Christensenellaceae was significantly higher in HCR rats than in LCR rats (Liu et al., 2015). Another similar ovariectomized model also validated that the microbial diversity and number of the Bacteroidetes phylum were significantly increased in LCR rats but unchanged in HCR rats without exercise intervention (Cox-York et al., 2015).

In traditional analysis methods, reductionist approaches are insufficient to comprehensively portray connections and regulation of complicated biological responses. The term *omics* implies a comprehensive (or global) assessment of a set of molecules. Several omics technologies exist, including genomics, epigenomics, transcriptomics, proteomics, metabolomics, and microbiomics, which are all well-developed and widely used in different fields (Hasin et al., 2017). Compared with a single omics type, multiomics can provide different perspectives on the flow of information underlying phenotypes, physiological responses, and disease development.

In this study, we examined the effects of intrinsic high exercise capacity (HEC) and low exercise capacity (LEC) on physical activity performance and on physiological and biochemical data using a multiomics approach. Our results could provide alternative perspectives on regulatory mechanisms for physiological adaptations and even on critical factors affecting health.

## Materials and Methods

### Animals and Group Design

**FIGURE 1:**
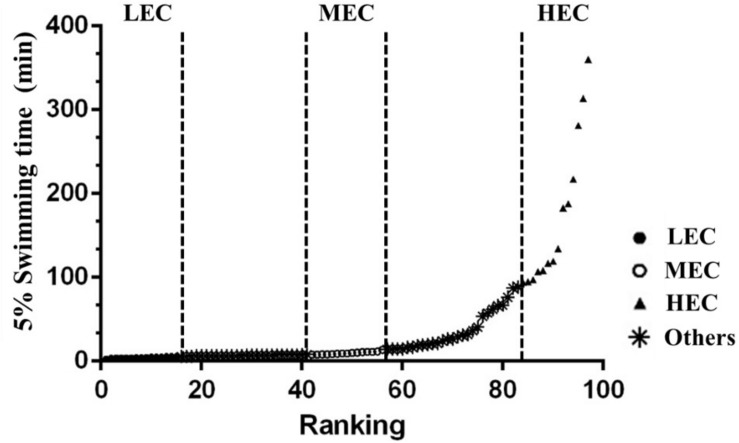
Capacity ranking from low to high for male mice (*n* = 100). The 15 lowest ranked, 15 medium ranked, and 15 highest ranked mice were categorized as low exercise capacity (LEC), medium exercise capacity (MEC), and high exercise capacity (HEC) mice for study experiments.

Male outbred ICR mice (7 weeks old) were purchased from a supplier accredited by the Association for Assessment and Accreditation of Laboratory Animal Care (BioLASCO, Yi-Lan, Taiwan). Experimental animals were maintained in the animal room at the National Taiwan Sport University (NTSU), and all procedures were approved by the Institutional Animal Care and Use Committee of the NTSU (IACUC-10103). All animals were maintained at 23 ± 2°C (room temperature) and 60 ± 5% humidity and were fed a standard laboratory diet (Laboratory Rodent Diet #5001, PMI Nutrition International, Brentwood, MO, United States) and distilled water *ad libitum*. Mice were acclimated to the environment for 1 week before experiments were conducted. Then, all 100 mice, without artificial selection population as F0 generation, performed an exhaustive swimming test, with 5% body weight (BW) loading on the tail. After the test, the mice were categorized into three groups on the basis of the exhaustive swimming time: LEC (15 lowest capacity mice), medium exercise capacity (MEC; 15 medium capacity mice), and HEC (15 highest capacity mice) (Figure 1).

### Exhaustive Swimming Exercise

Mice were individually placed in a columnar swimming pool (65 cm in height and 20 cm in diameter) with a 40-cm water depth maintained at 27 ± 1°C. A weight equivalent to 5% of the mouse’s BW was attached to the base of the tail, and for each mouse, endurance was measured as the swimming time recorded from the beginning of the time in the pool to exhaustion. The swimming period was defined as time spent floating, struggling, and making necessary movements until exhaustion – as indicated by evident uncoordinated movements and failure to swim to the water surface within 7 s (Huang et al., 2012).

### Forelimb Grip Strength

Forelimb grip strength was assessed 1 week after the exhaustive swimming test. A low-force testing system (Model-RX-5, Aikoh Engineering, Nagoya, Japan) was used to measure the absolute forelimb grip strength of mice, as previously described (Huang et al., 2012). Tensile force was measured using a force transducer equipped with a metal bar (2 mm in diameter and 7.5 cm in length) for each mouse. The maximal force (g) recorded was used as an indicator of absolute grip strength.

### Fatigue-Associated Biochemical Indices

Fatigue-associated biochemical indices were assessed 1 week after the forelimb grip strength test. Blood samples were collected immediately after mice swam for 15 min. After centrifugation at 1,500 × *g* for 10 min at 4°C, serum was analyzed on an automatic analyzer (Hitachi 7060, Hitachi, Tokyo, Japan). Biochemical variables, namely lactate, ammonia, blood urea nitrogen (BUN), glucose, lactic dehydrogenase (LDH), creatinine kinase (CK), aspartate aminotransferase (AST), and alanine aminotransferase (ALT), were evaluated as indices of exercise fatigue, as detailed previously (Huang et al., 2012; Wang et al., 2012; Wu et al., 2013).

### Oral Glucose Tolerance Test

An oral glucose tolerance test (OGTT) was conducted 1 week after assessment of fatigue-associated biochemical indices. Mice were fasted for 14 h before the OGTT. An oral gavage of glucose (2.5 g/kg BW) was administered, and blood samples were collected at 0, 15, 30, 60, and 120 min and analyzed using a glucometer, Roche Accu-Chek^®^ Comfort Curve (Roche Diagnostics, Indianapolis, IN, United States).

### Blood Biochemical Assessments

At the end of the final experiment, all mice were killed by 95% CO_2_ asphyxiation at an optimal flow rate for CO_2_ displacement of 15% chamber volume per minute; then, their blood was withdrawn through a cardiac puncture after an 8-h fas. The blood was centrifuged at 1,500 × *g* for 10 min at 4°C, and the serum was used to assess the levels of AST, ALT, LDH, albumin, total protein (TP), BUN, alkaline phosphatase (Alk-P), creatinine, CK, uric acid (UA), total cholesterol (TC), triacylglycerol (TG), and glucose using the autoanalyzer (Hitachi 7060, Tokyo, Japan).

### Tissue Sample Preparation

At the end of the study, the key tissues and organs – the liver, muscles (gastrocnemius and soleus muscles), kidney, heart, lung, epididymal fat pad (EFP), and brown adipose tissue (BAT) – were carefully removed and weighed. The total weight and specific tissue weights relative to individual BW (%) were recorded. Then, these tissues were immediately stored in liquid nitrogen, and the soleus muscle was collected separately for further proteomics and transcriptomics analyses.

### Bacterial DNA Extraction and 16S rRNA Sequencing

Fecal samples were collected 1 week after the OGTT. Mice in each group were housed in a metabolic cage with food and water calculated to produce individual feces. The feces were immediately stored at −80°C for bacterial DNA extraction. Feces (500 mg) were homogenized (MagNA Lyser System; Roche, Basel, Switzerland) in ASL buffer, and DNA was extracted directly using the QIAamp DNA Stool Mini Kit (Qiagen, Germany) according to the manufacturer’s instructions. Extracted DNA was stored at −80°C before 16S rRNA sequencing. The hypervariable V3–V5 region of the bacterial 16S rRNA gene was amplified through polymerase chain reaction (PCR) with barcoded universal primers V3-357F (forward primer; 5′-CCTA TCCCCTGTGTGCCTTGGCAGTCTCAG**CCTACGGGAGGCA GCAG**-3′) and V5-926R (reverse primer; 5′-CCATCTCATCCC TGCGTGTCTCCGACTCAG NNNNNN**CCGTCAATTCMTTT RAGT**-3′). The underlined sequences are the 454 FLX sequencing primers, and the bold letters denote the universal 16S rRNA primers. The barcode within the primer is denoted by Ns. The regions of the 16S rRNA gene were amplified using FastStart HiFi Polymerase (Roche, Basel, Switzerland). The sequencing reaction was performed in a 9700 thermal cycler (Applied Biosystems, Foster, CA, United States) at 94°C for 4 min, followed by 40 cycles at 94°C for 15 s, 50°C for 45 s, and 72°C for 1 min, then held at 72°C for 8 min, and finally maintained at 4°C until use. The presence of amplicons was confirmed through 1.5% agarose gel electrophoresis. PCR amplicons were purified using the Agencourt AMPure XP Reagent (Beckman Coulter^TM^, Pasadena, CA, United States) and quantified using Agilent Bioanalyzer (Agilent Technologies, Palo Alto, CA, United States). Equimolar amounts of PCR amplicons were mixed in a single tube. The purified amplicon mixtures were sequenced on the GS Junior System (454 Life Sciences, a Roche Company, Branford, CT, United States) according to the protocols recommended by the manufacturer. The sequences were further analyzed using a Ribosomal Database Project Naive Bayesian rRNA Classifier (version 2.5) to categorize the microbiota and investigate the relative proportions of microbiota existing in an indicated sample.

### Soleus Muscle MicroRNA TaqMan Low-Density Array Analysis

Total RNA was isolated using the TRI reagent (Thermo Scientific, San Jose, CA, United States) for further miRNA expression profiling. Total RNA was quantified using NanoDrop ND-1000 (Thermo Scientific, San Jose, CA, United States), and RNA integrity (RNA integrity number ≥ 6) was verified using the RNA 6000 Nano kit (Agilent Technologies, Palo Alto, CA, United States). The miRNA expression profile in the soleus muscle of mice in each capacity group was analyzed using the TaqMan Low-Density Array (TLDA) Rodent MicroRNA Cards (version 3A and B; Applied Biosystems, Foster, CA, United States). Each card contains 375 precoated rodent miRNA targets, all cataloged in the miRBase database, with three endogenous controls: Mamm U6, U87, and Y1. In this study, U87 was used as the endogenous normalizer. Total RNA (100 ng) was reverse-transcribed using Megaplex RT primer Pools A and B (381 stem-looped primers per pool) and the TaqMan MicroRNA Reverse Transcription kit (Applied Biosystems, Foster, CA, United States). The preamplified product was diluted with the TaqMan Universal PCR master mix (Applied Biosystems, Foster, CA, United States) and deionized distilled water, and it was loaded into one of the eight fill ports on the TLDA microfluidic card. The card was centrifuged for 1 min at 331 × *g* to distribute samples to multiple wells connected to the fill ports and was then sealed to prevent well-to-well contamination. All cards were processed and analyzed on a 7900 HT Real-Time PCR System (Applied Biosystems, Foster, CA, United States). Real-time RT-PCR data analysis was performed using the RQ Manager (version 1.2.1; Applied Biosystems, Foster, CA, United States) and Partek Genomic Suite (version 6.6). The expression level was calculated using the comparative Ct (ΔΔCt) method and was further analyzed by comparing the fold change relative to HEC and LEC. The results of the TLDA analysis were converted into a graphic display heat map based on hierarchical clustering using DataAssist (version 2.0).

### Soleus Muscle Proteomics Analysis

Soleus muscle protein samples (50 μg) were applied to the gel in triplicate, and the sizes of proteins were visualized by staining with Coomassie Brilliant Blue G-250 (Bio-Rad, Hercules, CA, United States). After electrophoresis gel lanes were split into 10 equal fractions, the slices were destained through repeated washing in a solution of 25 mM ammonium bicarbonate and 50% (V/V) acetonitrile (1:1) until the protein bands were invisible. After completely drying them on Speed-Vac (Thermo Electron, Waltham, MA, United States), the proteins in the gel fragments were then subjected to reduction and cysteine alkylation reactions to irreversibly break the disulfide bridges in the proteins. For reduction, each gel piece was rehydrated with 2% (V/V) β-mercaptoethanol in 25 mM ammonium bicarbonate and incubated at room temperature for 20 min in the dark. Cysteine alkylation was performed by adding an equal volume of 10% (V/V) 4-vinylpyridine in 25 mM ammonium bicarbonate and 50% (V/V) acetonitrile for 20 min. The samples were then washed by soaking them in 1 mL of 25 mM ammonium bicarbonate for 10 min. After drying on Speed-Vac for 20 min, in-gel trypsin digestion was performed by incubating the samples with 100 ng of modified trypsin (Promega, Mannheim, Germany) in 25 mM ammonium bicarbonate at 37°C overnight. The supernatant of the tryptic digest was transferred to an Eppendorf tube. For extraction of the remaining peptides from the gel, 25 mM ammonium bicarbonate and 50% (V/V) acetonitrile were added, the samples were incubated for 10 min, and then the solution was collected. The resulting digests were dried in Speed-Vac and stored at −20°C. Each cryo-stored tryptic digest was resuspended in 30 μL of 0.1% (V/V) formic acid and analyzed using an online nanoAcquity ultra-performance liquid chromatography system (Waters, Manchester, United Kingdom) coupled to a hybrid linear ion trap Orbitrap (LTQ-Orbitrap Discovery) mass spectrometer with a nanoelectrospray ionization source (Thermo Scientific, San Jose, CA, United States). The eluted peptides were ionized with a spray of 2.33 kV and placed in the mass spectrometer. Mass spectrometric data were obtained using the data-dependent acquisition method, in which one full mass spectrometry survey scan (*m*/*z* 200–1,500) at a high resolution of 30,000 full width at half maximum width was followed by tandem MS (*m*/*z* 200–1,500) of the six most intense multiple-charged ions. A protein was identified when at least two unique peptides were matched with an Xcorr score of >2.5 for each peptide. The false discovery rate (≤1%) obtained from the search was compared against the decoy database to identify the protein.

### Statistical Analysis

Statistical analyses were performed using SPSS (version 18.0; SPSS, Chicago, IL, United States). Data are expressed as mean ± standard deviation (SD). Significant differences in weight, diet, body composition, biochemical data, and physical activity performance among HEC, MEC, and LEC mice were calculated using one-way analysis of variance (ANOVA) and Duncan’s test, and *p* < 0.05 was considered significant. In addition, an independent *t-*test was used to compare the intestinal microbiota, transcriptome, and proteome of soleus muscle between HEC and LEC mice, and *p* < 0.05 (^∗^) was considered statistically significant.

## Results

### Intrinsic Aerobic Endurance and Grip Strength Performance

**FIGURE 2:**
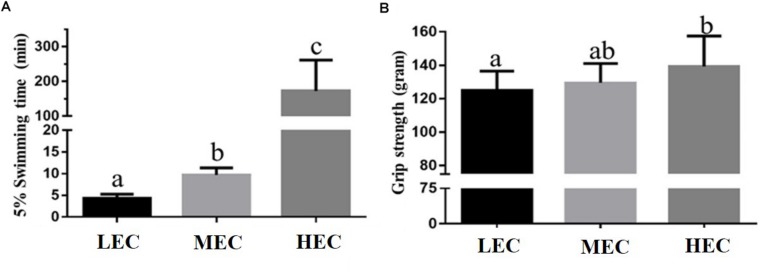
Effect of exercise capacity on **(A)** exhaustive swimming time and **(B)** forelimb grip strength. Data are presented as mean ± standard deviation for 15 mice per group. Different letters (a,b,c) indicate a significant difference at *p* < 0.05 using one-way ANOVA.

Mice (*n* = 100) performed an exhaustive swimming test under 5% BW loading. Intrinsic aerobic endurance was ranked according to the exhaustive swimming exercise performance of mice (Figure 1). Most mice were exhausted within 100 min, but a few mice demonstrated extreme endurance capacity and swam for >200 min. On the basis of their performance, mice were divided into three experimental groups: LEC (bottom 0–15% performers), MEC (intermediate 42.5–57.5% performers), and HEC (top 85–100% performers) groups. Each group contained 15 mice, all of whom underwent additional physical tests. The exhaustive swimming times of LEC, MEC, and HEC mice were 4.35 ± 0.23, 9.72 ± 0.42, and 172.4 ± 23.79 min, respectively (Figure 2A). Thus, the exhaustive swimming exercise performance of HEC mice significantly exceeded that of LEC mice by 38.6-fold (*p* < 0.001). In addition, the grip strength of HEC mice (139.2 ± 5.1 g) was higher than that of LEC (124.9 ± 3.2 g) and MEC (129.5 ± 3.2 g) mice (*p* = 0.032 and *p* = 0.038, respectively; Figure 2B).

### BW, Food Uptake, and Tissue Weight

Table 1 lists the BW, daily food intake, and tissue weight of LEC, MEC, and HEC mice. BW, diet, and water intake did not significantly differ among the groups. Therefore, differences in exercise performance were not attributable to nutritional supplementation but to other adaptive physiological factors. In addition, only the muscle weight (particularly the weight of gastrocnemius and soleus muscles) was significantly different between LEC and HEC mice (Table 1): It was 7.7% higher in HEC mice than in LEC mice (*p* = 0.042).

**TABLE 1:** Effect of different exercise capacities on general characteristics.

**Characteristic**	**LEC**	**MEC**	**HEC**	**Trend analysis**
Initial BW (g)	35.3 ± 0.7	34.8 ± 0.7	34.4 ± 0.6	0.9655
Final BW (g)	39.5 ± 0.7	40.1 ± 0.9	39.6 ± 0.5	0.2490
Food intake (g/day)	7.9 ± 0.4	7.5 ± 0.3	7.8 ± 0.3	0.9344
Water intake (mL/day)	11.8 ± 0.4	10.9 ± 0.4	10.9 ± 0.2	0.2374
Liver (g)	2.27 ± 0.07	2.22 ± 0.05	2.25 ± 0.07	0.8609
Muscle (g)	0.39 ± 0.02^a^	0.40 ± 0.01^ab^	0.42 ± 0.01^b^	0.0504
Kidney (g)	0.71 ± 0.02	0.70 ± 0.01	0.70 ± 0.02	0.8611
Heart (g)	0.26 ± 0.01	0.25 ± 0.01	0.25 ± 0.01	0.9842
Lung (g)	0.21 ± 0.01	0.21 ± 0.01	0.22 ± 0.01	0.9729
EFP (g)	0.29 ± 0.03	0.26 ± 0.02	0.27 ± 0.01	0.9388
BAT (g)	0.09 ± 0.001^a^	0.11 ± 0.006^b^	0.09 ± 0.002^a^	0.3432

*Muscle mass includes both gastrocnemius and soleus muscles in the back part of the lower legs. LEC, low exercise capacity; MEC, medium exercise capacity; HEC, high exercise capacity; BW, body weight; EFP, epididymal fat pad; BAT, brown adipocyte tissue. Data are the mean ± standard error measurement for *n* = 10 mice in each group. Values in the same row with different superscript letters (a, b) differ significantly, *p* < 0.05, by one-way ANOVA.*

### Clinical Biochemistry

**FIGURE 3:**
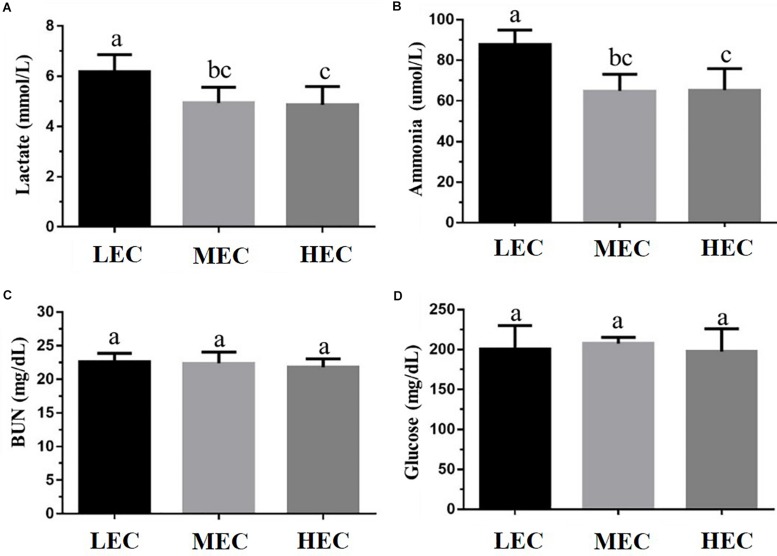
Effect of exercise capacity on fatigue-related biomarkers, namely serum **(A)** lactate, **(B)** ammonia, **(C)** blood urea nitrogen (BUN), and **(D)** glucose level, after the acute exercise challenge. Data are presented as mean ± standard deviation for 15 mice in each group. Different letters (a,b,c) indicate a significant difference at *p* < 0.05 using one-way ANOVA.

**FIGURE 4:**
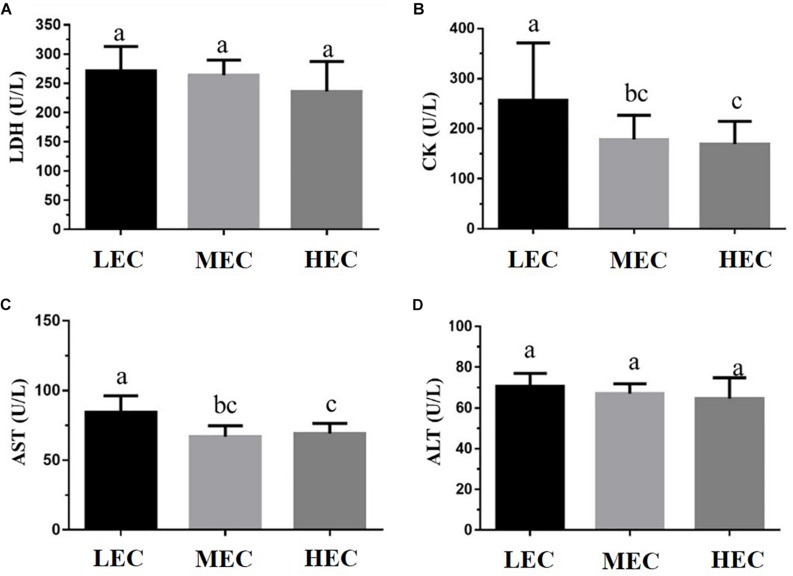
Effect of exercise capacity on injury-related markers, namely serum **(A)** lactic dehydrogenase (LDH), **(B)** creatinine kinase (CK), **(C)** aspartate aminotransferase (AST), and **(D)** alanine transaminase (ALT) levels, after the acute exercise challenge. Data are presented as mean ± standard deviation for 15 mice in each group. Different letters (a,b,c) indicate a significant difference at *p* < 0.05 using one-way ANOVA.

We assessed fatigue-related (lactate, ammonia, BUN, and glucose) and injury-related (LDH, CK, AST, and ALT) biomarkers after the acute exercise challenge and evaluated biochemical data at the end of the study. Lactate and ammonia levels in HEC and MEC mice were significantly lower than those in LEC mice (*p* < 0.05; Figure 3). Regarding injury-related biomarkers, HEC and MEC mice had significantly lower CK and AST levels than LEC mice (*p* < 0.05; Figure 4). The remaining indicators, namely BUN, glucose, LDH, and ALT, did not differ significantly among the three groups (*p* > 0.05).

Table 2 presents the biochemical data assessed at the end of the study. Compared with LEC mice, HEC and MEC mice demonstrated significantly higher serum albumin levels but significantly lower BUN levels (both *p* < 0.05). Although ALT, LDH, and Alk-P levels were significantly different between the three groups, no significant difference was noted between HEC and LEC mice (*p* > 0.05). Similarly, AST, TP, creatinine, CK, UA, TC, TG, and Glu levels did not differ significantly among the three groups (*p* > 0.05).

**TABLE 2:** Effect of different exercise capacity on biochemical analysis at the end of treatment.

**Parameter**	**LEC**	**MEC**	**HEC**	**Trend analysis**
AST (U/L)	70 ± 4	81 ± 4	73 ± 3	0.545
ALT (U/L)	44 ± 3^a^	61 ± 4^b^	54 ± 2^ab^	0.022
LDH (U/L)	246 ± 12^a^	387 ± 27^b^	333 ± 24^ab^	0.034
Albumin (g/dL)	3.2 ± 0.03^a^	3.3 ± 0.04^b^	3.4 ± 0.06^b^	0.046
TP (g/dL)	4.6 ± 0.1	4.6 ± 0.1	4.6 ± 0.1	0.757
BUN (mg/dL)	23.5 ± 0.7^b^	20.4 ± 0.7^a^	21.1 ± 0.4^a^	0.003
Alk-P (U/L)	38.4 ± 1.8^a^	47.9 ± 3.3^b^	45.6 ± 2.6^ab^	0.017
Creatinine (mg/dL)	0.13 ± 0.01	0.13 ± 0.01	0.12 ± 0.01	0.590
CK (U/L)	286 ± 41	307 ± 37	285 ± 46	0.911
UA (mg/dL)	1.5 ± 0.1	1.7 ± 0.1	1.6 ± 0.1	0.646
TC (mg/dL)	126 ± 6	137 ± 5	136 ± 4	0.301
TG (mg/dL)	141 ± 9	151 ± 9	139 ± 9	1.000
Glu (mg/dL)	157 ± 6	152 ± 7	159 ± 5	0.870

*Data are mean ± standard error measurement for *n* = 10 mice in each group. Different superscript letters (a, b) indicate significant difference (*p* < 0.05) by one-way ANOVA.*

### OGTT

**FIGURE 5:**
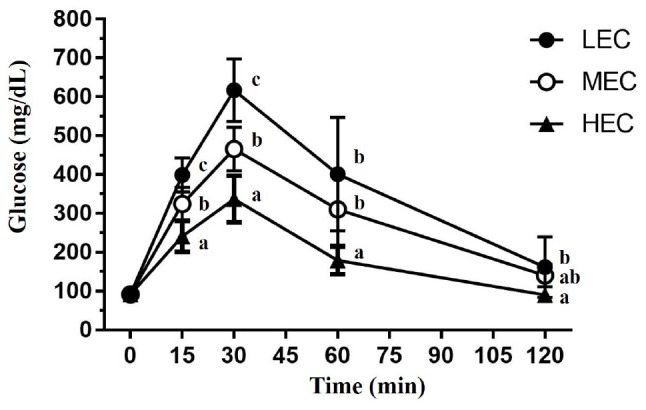
The oral glucose tolerance test was performed in mice with various exercise capacities at the same glucose dosage (2 g/kg) after 14 h of fasting. The indicated sampling times (0, 15, 30, 60, and 120 min) were plotted as the tolerance curve. Data are presented as mean ± standard deviation for 15 mice in each group. Different letters (a,b,c) indicate a significant difference at *p* < 0.05 using one-way ANOVA.

After the administration of glucose (2.5 g/kg) through oral gavage, blood was individually collected for glucose measurement at 0, 15, 30, 60, and 120 min (Figure 5). At the beginning (0 min), glucose levels did not differ significantly among the three groups (*p* > 0.05). However, at 15 and 30 min, glucose levels were significantly higher in LEC mice than in MEC and HEC mice (*p* = 0.024 and *p* < 0.001, respectively). At 60 min, LEC and MEC mice did not exhibit significantly different glucose levels; however, HEC mice had significantly lower glucose levels than did LEC and MEC mice (*p* = 0.004 and *p* = 0.012, respectively). Finally, at 120 min, LEC mice exhibited 2.01-fold higher glucose levels than HEC mice (*p* < 0.001).

### Gut Microbiota

**FIGURE 6:**
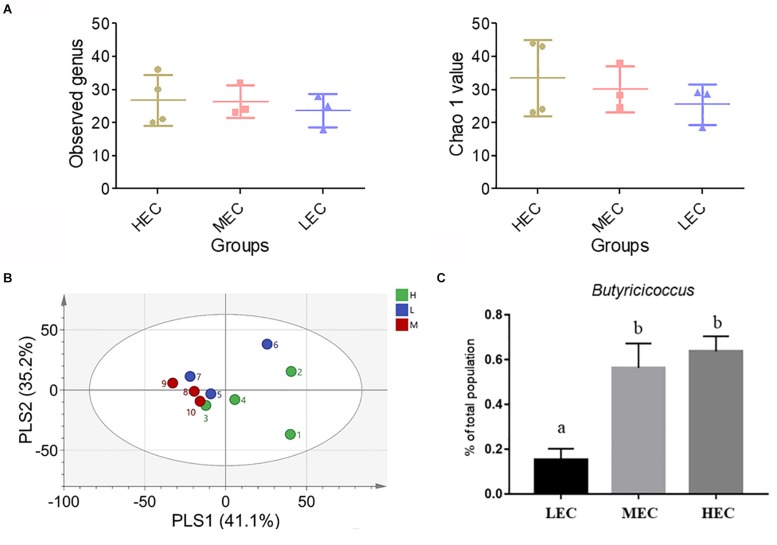
Effect of exercise capacity on **(A)** partial least squares discriminant analysis (PLSDA) of genus level, **(B)** alpha-diversity indexes of the gut microbiota composition, and **(C)** *Butyricicoccus*. Different letters (a,b) indicate a significant difference at *p* < 0.05 using one-way ANOVA.

A partial least squares discriminant analysis showed that mice clustered into relatively distinct groups based on LEC, MEC, and HEC mice (Figure 6A). Alpha-diversity indexes of richness (Observed and Chao1) were slightly increased in HEC mice compared with LEC mice (Figure 6B). The gut microbiota of HEC and LEC mice altered considerably. Nine phyla were detected: Actinobacteria, Bacteroidetes, Crenarchaeota, Cyanobacteria/Chloroplast, Deferribacteres, Firmicutes, Proteobacteria, Tenericutes, and TM7; the ratios of these phyla did not differ significantly between HEC and LEC mice. Furthermore, 13 classes were noted: Actinobacteria, Alphaproteobacteria, Bacilli, Bacteroidia, Betaproteobacteria, Chloroplast, Clostridia, Deferribacteres, Deltaproteobacteria, Erysipelotrichia, Gammaproteobacteria, Moliciches, and Thermoprotei. The proportion of Betaproteobacteria was significantly lower in HEC mice than in LEC mice (*p* = 0.04). Next, 14 orders were identified: Anaeroplasmatales, Bacillales, Bacteroidales, Burkholderiales, Clostridiales, Coriobacteriale, Deferribacterales, Desulfovibrionales, Enterobacteriales, Erysipelotrichales, Lactobacillales, Rhizobiale, Rhodospirillales, and Sphingomonadales. Compared with LEC mice, HEC mice exhibited a significantly lower number of Burkholderiales and Rhizobiales (*p* = 0.034 and 0.017, respectively) and a significantly higher number of Deferribacterales (*p* = 0.025). Of the 21 identified families, the number of Anaeroplasmataceae and Sutterellaceae was significantly lower in HEC mice than in LEC mice (*p* = 0.025 and 0.035). Of the 49 identified genera, the number of *Anaeroplasma*, *Anaerovorax*, *Erysipelotrichia*, *Gemmiger*, and *Parasutterella* was significantly lower in HEC mice than in LEC mice (*p* = 0.030, 0.015, 0.007, 0.018, and 0.04, respectively); however, the number of *Butyricicoccus* was significantly higher in HEC mice than in LEC mice (*p* = 0.038; Figure 6C).

### Soleus Muscle miRNA Profiles

The 47 miRNA expression profiles of the soleus muscles, revealed using TLDA, varied among the three groups (Supplementary Figure 1). Compared with LEC mice, HEC mice exhibited significant changes (27.13–39.44-fold) in several miRNAs (Table 3). Seven miRNAs, namely miR-383, miR-107, miR-30b, miR-669m, miR-191, miR-218, and miR-224, were significantly upregulated in HEC mice (Table 4). Thus, a set of candidate miRNAs regulated in soleus muscles could be used as potential diagnostic biomarkers of intrinsic exercise capacities.

**TABLE 3:** Effect of different exercise capacities on miRNA expression.

**miR ID**	***p***-**value**	**Mean ratio (HEC vs. LEC)**	**Fold change (HEC vs. LEC)**
mmu-miR-878-3p-002541	1.77*E*−02	27.13	27.13
mmu-miR-383-4381093	1.18*E*−03	18.43	18.43
mmu-miR-719-001673	2.40*E*−01	13.50	13.50
mmu-miR-672-4395438	2.52*E*−01	12.22	12.22
mmu-miR-423-5p-4395451	2.54*E*−01	12.11	12.11
hsa-miR-183#-002270	2.76*E*−01	10.80	10.80
mmu-miR-183-4395380	1.51*E*−01	10.19	10.19
mmu-miR-466b-3-3p-002500	6.42*E*−02	10.12	10.12
mmu-miR-543-4395487	1.69*E*−01	9.77	9.77
hsa-miR-338-000548	2.92*E*−01	9.36	9.36
mmu-miR-137-4373301	7.21*E*−02	9.22	9.22
mmu-miR-298-4395728	3.61*E*−02	8.67	8.67
mmu-miR-34c-4373036	1.16*E*−01	7.23	7.23
mmu-miR-466h-4395646	6.52*E*−02	7.00	7.00
mmu-miR-465b-5p-4395615	3.16*E*−01	6.73	6.73
rno-miR-347-4381114	2.75*E*−01	6.49	6.49
mmu-miR-743a-4395599	2.54*E*−01	6.39	6.39
mmu-miR-330-4395341	1.77*E*−01	6.00	6.00
mmu-miR-181A-2#-002687	1.19*E*−01	5.58	5.58
mmu-miR-182-4395729	1.99*E*−01	5.52	5.52
mmu-miR-1962-121173_mat	9.72*E*−02	5.47	5.47
mmu-miR-362-5p-002614	5.55*E*−02	5.25	5.25
mmu-miR-1193-002794	3.78*E*−01	4.83	4.83
mmu-miR-455-4395585	3.82*E*−02	4.74	4.74
mmu-miR-107-4373154	3.68*E*−01	4.55	4.55
mmu-miR-450a-5p-4395414	1.88*E*−01	4.20	4.20
hsa-miR-9#-002231	7.62*E*−02	4.00	4.00
mmu-miR-30b#-002498	2.54*E*−03	3.98	3.98
rno-miR-207-4381096	1.30*E*−01	3.92	3.92
mmu-miR-669m-121190_mat	4.72*E*−02	3.92	3.92
mmu-miR-294-4373326	2.61*E*−01	3.84	3.84
mmu-miR-138#-002554	2.14*E*−01	3.78	3.78
mmu-miR-191#-002576	3.08*E*−01	3.68	3.68
rno-miR-20b-001326	7.61*E*−02	3.60	3.60
mmu-miR-20b-4373263	1.26*E*−01	3.54	3.54
mmu-miR-10a-4373153	1.97*E*−01	3.53	3.53
mmu-miR-433-4373205	1.89*E*−01	3.47	3.47
mmu-miR-1896-121128_mat	7.14*E*−02	3.41	3.41
mmu-miR-218-1#-002552	2.00*E*−01	3.31	3.31
mmu-miR-202-3p-4373311	1.86*E*−02	3.21	3.21
mmu-miR-501-3p-4381069	7.69*E*−02	3.10	3.10
mmu-miR-470-4395718	2.66*E*−01	3.10	3.10
mmu-miR-434-5p-4395711	4.27*E*−01	3.09	3.09
mmu-miR-211-4373315	3.44*E*−01	3.08	3.08
rno-miR-224-4373187	8.47*E*−02	3.04	3.04
rno-miR-1-4395765	2.90E−01	0.22	−4.65
mmu-miR-1188-002866	2.50E−01	0.03	−39.44

*The significant difference (*p* < 0.05) for high exercise capacity (HEC) and low exercise capacity (LEC) groups was compared using the *t*-test.*

**TABLE 4:** Soleus miRNA molecules with significant difference related to intrinsic exercise capacity.

**miRNA molecules**	**Biological functions**	**Fold change (HEC/LEC)**	***p*-value**
miR-383	(1) Testosterone and progesterone regulation, (2) spermatogenesis, and (3) steroidogenesis	18.43	1.18*E*−03
miR-107	Insulin sensitivity	4.55	3.68*E*−01
miR-30b	Role of regeneration	3.98	2.54*E*−03
miR-669m	Adaptation during toxic metabolites accumulation	3.92	4.72*E*−02
miR-191	Adipogenesis inhibition	3.68	3.08*E*−01
miR-218	Vascular organization	3.31	2.00*E*−01
miR-224	Adipogenesis regulation (fatty acid metabolism)	3.04	8.47*E*−02

*The significant difference (*p* < 0.05) for high exercise capacity (HEC) and low exercise capacity (LEC) groups was compared by *t*-test.*

### Soleus Muscle Proteomics Analysis

The soleus muscle protein profiles of LEC and HEC mice were analyzed using LC-MS/MS; next, various soleus muscle proteins were compared between these mice (Table 5). We detected 97 soleus proteins in LEC and HEC mice; of these, 8 were common and 89 were significantly regulated proteins in LEC and HEC mice. Among them, the levels of 79 and 10 proteins were significantly increased and decreased, respectively, in HEC mice compared with LEC mice. These major pathways between LEC and HEC mice were generated using ingenuity pathway analysis (IPA), where *p* < 0.05. In Supplementary Figure 2, the length of the bar only indicates that the differentially expressed proteins are related to this pathway but not that the pathway is either upregulated or downregulated. Soleus muscle proteins related to biofunctions and physiological metabolic pathways in HEC and LEC mice underwent significant changes related to (a) muscle contraction, (b) cardiovascular disease and organismal injury, (c) skeletal and muscle function, (d) carbohydrate metabolism, (e) muscle cell morphology, and (f) organ morphology.

**TABLE 5:** Differential expression for protein molecules in soleus between low exercise capacity (LEC) and high exercise capacity (HEC) groups.

**Protein name**	**Protein-ID**	**Gene-ID**	**HEC (mean ± SE)**	**LEC (mean ± SE)**	***p*-value**
Myosin, heavy polypeptide 6, cardiac muscle, alpha	Q2TAW4	Q2TAW4	107.87 ± 26.34	10.53 ± 8.55	0.01259
Calsequestrin	Q6P3C3	Q6P3C3	28.3 ± 3.34	16.57 ± 2.29	0.02739
Calsequestrin-1	O09165	CASQ1	28.3 ± 3.34	16.57 ± 2.29	0.02739
Beta-enolase	P21550	ENOB	12.88 ± 1.82	5.19 ± 0.97	0.00971
Cytochrome b–c1 complex subunit 1, mitochondrial	Q9CZ13	QCR1	3.84 ± 0.56	1.62 ± 0.71	0.04955
Putative uncharacterized protein	Q3THM1	Q3THM1	3.84 ± 0.56	1.62 ± 0.71	0.04955
Putative uncharacterized protein	Q3TIC8	Q3TIC8	3.84 ± 0.56	1.62 ± 0.71	0.04955
Putative uncharacterized protein	Q3UIQ2	Q3UIQ2	9.02 ± 1.44	1.77 ± 1.77	0.01918
Putative uncharacterized protein	Q3TX47	Q3TX47	2.18 ± 0.79	0 ± 0	0.03254
Adenylosuccinate synthetase isozyme 1	P28650	PURA1	2.37 ± 0.46	0 ± 0	0.00222
Adenylosuccinate synthetase isozyme 1	J3QN31	J3QN31	2.37 ± 0.46	0 ± 0	0.00222
Glucose-6-phosphate isomerase	P06745	G6PI	10.88 ± 2.67	3.96 ± 0.42	0.04274
Putative uncharacterized protein	Q3U6X6	Q3U6X6	5.1 ± 1.95	0 ± 0	0.03993
Phosphoglucomutase-1	A2CEK3	A2CEK3	5.1 ± 1.95	0 ± 0	0.03993
Myosin light chain 3	P09542	MYL3	1.98 ± 0.75	5.83 ± 1.35	0.04648
Synaptopodin 2	B2RY03	B2RY03	6.48 ± 0.7	5.07 ± 0.92	0.26857
Synaptopodin-2	E9Q1U2	E9Q1U2	6.48 ± 0.7	5.07 ± 0.92	0.26857
Serum deprivation-response protein	Q63918	SDPR	1.19 ± 0.41	0 ± 0	0.02615
2-Oxoglutarate dehydrogenase, mitochondrial	Q60597	ODO1	10.05 ± 1.85	14.69 ± 1.04	0.07145
Moesin	P26041	MOES	0 ± 0	0.37 ± 0.37	0.35592
Nucleoside diphosphate kinase A	P15532	NDKA	0 ± 0	0.37 ± 0.37	0.35592
Nucleoside diphosphate kinase B	Q01768	NDKB	0 ± 0	0.37 ± 0.37	0.35592
Putative uncharacterized protein	Q3TZQ2	Q3TZQ2	0 ± 0	0.37 ± 0.37	0.35592
Radixin	P26043	RADI	0 ± 0	2.23 ± 1.01	0.06861
6-Phosphofructokinase, muscle type	P47857	K6PF	2.05 ± 0.75	0 ± 0	0.03472
Polymerase I and transcript release factor	O54724	PTRF	3.28 ± 0.44	1.2 ± 0.7	0.04571
Triosephosphate isomerase	P17751	TPIS	5.18 ± 1.06	0.97 ± 0.56	0.01249
Synaptopodin 2-like protein	Q8BWB1	SYP2L	3.32 ± 0.21	1.55 ± 0.58	0.02826
Synaptopodin 2-like protein	B2RQK7	B2RQK7	3.32 ± 0.21	1.55 ± 0.58	0.02826
Synaptopodin 2-like protein	D3YU08	D3YU08	3.32 ± 0.21	1.55 ± 0.58	0.02826
Putative uncharacterized protein	Q3UDU4	Q3UDU4	8.93 ± 2.6	2.12 ± 0.71	0.04491
Uncharacterized protein	M0QWZ0	M0QWZ0	17.19 ± 4.16	6.94 ± 0.38	0.04961
PDZ and LIM domain protein 5	Q9CRA2	Q9CRA2	1.74 ± 0.59	0 ± 0	0.02664
Nucleoside diphosphate kinase	E9PZF0	E9PZF0	2.31 ± 0.29	0.62 ± 0.62	0.04843
Aspartate aminotransferase, mitochondrial	P05202	AATM	3.98 ± 1.62	0 ± 0	0.04945
Collagen alpha-1(XV) chain	O35206	COFA1	1.24 ± 0.43	0 ± 0	0.02871
Collagen alpha-1(XV) chain	A2AJY2	A2AJY2	1.24 ± 0.43	0 ± 0	0.02871
Collagen alpha-1(XV) chain	A2AJY7	A2AJY7	1.24 ± 0.43	0 ± 0	0.02871
Ryanodine receptor 1	E9PZQ0	RYR1	1.67 ± 0.59	0 ± 0	0.02968
Ryanodine receptor 1	K3W4M2	K3W4M2	1.67 ± 0.59	0 ± 0	0.02968
Glyceraldehyde-3-phosphate dehydrogenase	P16858	G3P	22.26 ± 1.68	7.88 ± 0.83	0.00026
Very long-chain-specific acyl-CoA dehydrogenase, mitochondrial	P50544	ACADV	3.18 ± 0.55	0 ± 0	0.00119
Protein Mybpc1	D3YU50	D3YU50	23.96 ± 1.47	11.29 ± 3.15	0.01081
Protein Mybpc1	Q6P6L5	Q6P6L5	23.96 ± 1.47	11.29 ± 3.15	0.01081
Troponin T, fast skeletal muscle	A2A6H6	A2A6H6	23.17 ± 1.95	12.68 ± 1.01	0.00309
Troponin T, fast skeletal muscle	A2A6I8	A2A6I8	23.17 ± 1.95	12.68 ± 1.01	0.00309
Troponin T, fast skeletal muscle	A2A6J0	A2A6J0	23.17 ± 1.95	12.68 ± 1.01	0.00309
Troponin T, fast skeletal muscle	A2A6J1	A2A6J1	23.17 ± 1.95	12.68 ± 1.01	0.00309
Troponin T, fast skeletal muscle	J3QP61	J3QP61	23.17 ± 1.95	12.68 ± 1.01	0.00309
Phosphoglucomutase-1	Q9D0F9	PGM1	11.11 ± 1.02	6.84 ± 0.89	0.01973
Actin, alpha skeletal muscle	P68134	ACTS	132.76 ± 8.24	96.79 ± 6.47	0.0139
Alpha-actinin-3	O88990	ACTN3	32.2 ± 3.49	21.89 ± 1.75	0.03854
Actin, alpha cardiac muscle 1	P68033	ACTC	132.76 ± 8.24	96.79 ± 6.47	0.0139
Putative uncharacterized protein	Q3TG92	Q3TG92	132.76 ± 8.24	96.79 ± 6.47	0.0139
Putative uncharacterized protein	Q9CXK3	Q9CXK3	132.76 ± 8.24	96.79 ± 6.47	0.0139
LIM domain-binding protein 3	E9PYJ9	E9PYJ9	15.02 ± 1.72	9.84 ± 1.22	0.0494
L-Lactate dehydrogenase	G5E8N5	G5E8N5	11.76 ± 0.94	2.12 ± 0.71	0.00018
L-Lactate dehydrogenase A chain	P06151	LDHA	11.76 ± 0.94	2.12 ± 0.71	0.00018
Putative uncharacterized protein	Q3TI99	Q3TI99	11.76 ± 0.94	2.12 ± 0.71	0.00018
Collagen alpha-1(XV) chain	A2AJY5	A2AJY5	1.24 ± 0.43	0 ± 0	0.02871
MCG140784	Q792Z1	Q792Z1	31.39 ± 1.84	25.1 ± 1.58	0.04076
Try10-like trypsinogen	Q7M754	Q7M754	31.39 ± 1.84	25.1 ± 1.58	0.04076
Alpha-enolase	P17182	ENOA	2.11 ± 0.72	0 ± 0	0.02591
Krt6b protein	Q0VDR7	Q0VDR7	25.65 ± 3.96	4.94 ± 1.75	0.00306
60S ribosomal protein L7a	P12970	RL7A	2.63 ± 0.6	0 ± 0	0.00448
MCG18601	D3YVE6	D3YVE6	2.63 ± 0.6	0 ± 0	0.00448
Ribosomal protein L7A	Q5EBG5	Q5EBG5	2.63 ± 0.6	0 ± 0	0.00448
Ribosomal protein L7A	Q6P1A9	Q6P1A9	2.63 ± 0.6	0 ± 0	0.00448
Uncharacterized protein	D3YXT4	D3YXT4	2.63 ± 0.6	0 ± 0	0.00448
Uncharacterized protein	L7N202	L7N202	2.63 ± 0.6	0 ± 0	0.00448
Troponin T, fast skeletal muscle	Q9QZ47	TNNT3	15.56 ± 3.67	5.59 ± 0.51	0.03597
Transitional endoplasmic reticulum ATPase	Q01853	TERA	7.78 ± 1.31	4.11 ± 0.71	0.04918
Uncharacterized protein	D3YU93	D3YU93	1.54 ± 0.54	0 ± 0	0.02921
Uncharacterized protein	F6U2H0	F6U2H0	1.54 ± 0.54	0 ± 0	0.02921
Tropomyosin beta chain	P58774	TPM2	76.85 ± 19.02	15 ± 7.44	0.02316
M-protein	O55124	O55124	13.57 ± 2.5	2.39 ± 1.52	0.00882
Myomesin 2	Q14BI5	Q14BI5	13.57 ± 2.5	2.39 ± 1.52	0.00882
Putative uncharacterized protein	Q3UQS9	Q3UQS9	13.57 ± 2.5	2.39 ± 1.52	0.00882
Carbonic anhydrase 3	P16015	CAH3	9.75 ± 1.1	2.97 ± 1.45	0.00967
Creatine kinase M-type	P07310	KCRM	37.66 ± 4.36	7.89 ± 2.65	0.00112
Putative uncharacterized protein	Q9D6U7	Q9D6U7	37.66 ± 4.36	7.89 ± 2.65	0.00112
Myosin-binding protein C, fast-type	Q5XKE0	MYPC2	27.79 ± 2.85	13.96 ± 3.22	0.01815
6-Phosphofructokinase	Q99K08	Q99K08	2.05 ± 0.75	0 ± 0	0.03472
Pyruvate carboxylase	E9QPD7	E9QPD7	3.33 ± 1.17	0 ± 0	0.02909
Pyruvate carboxylase	G5E8R3	G5E8R3	3.33 ± 1.17	0 ± 0	0.02909
Pyruvate carboxylase	Q3T9S7	Q3T9S7	3.33 ± 1.17	0 ± 0	0.02909
Pyruvate carboxylase	Q3TCQ3	Q3TCQ3	3.33 ± 1.17	0 ± 0	0.02909
Pyruvate carboxylase, mitochondrial	Q05920	PYC	3.33 ± 1.17	0 ± 0	0.02909
Synaptopodin-2	D3YVV9	D3YVV9	1.41 ± 0.49	5.07 ± 0.92	0.01295
Collagen alpha-2(I) chain	Q01149	CO1A2	3.53 ± 0.49	5.77 ± 0.76	0.04821
Putative uncharacterized protein	Q3TU64	Q3TU64	3.53 ± 0.49	5.77 ± 0.76	0.04821
Collagen alpha-1(I) chain	P11087	CO1A1	8.24 ± 0.2	13.2 ± 1.33	0.01023
Microtubule-associated protein	E9PWC0	E9PWC0	0.38 ± 0.38	2.14 ± 0.29	0.01021
Microtubule-associated protein	E9PZ43	E9PZ43	0.38 ± 0.38	2.14 ± 0.29	0.01021
Putative uncharacterized protein	Q8BIZ5	Q8BIZ5	31.15 ± 1.84	48.14 ± 5.33	0.0235
Terminal uridylyltransferase 4	B2RX14	TUT4	31.15 ± 1.84	48.14 ± 5.33	0.0235
Terminal uridylyltransferase 4	A2A8R7	A2A8R7	31.15 ± 1.84	48.14 ± 5.33	0.0235

*Data are mean ± standard error (SE) measurement for *n* = 4 mice per group. The significant difference (*p* < 0.05) for high and low exercise capacity groups was compared using the *t*-test.*

## Discussion

For the 100 mice that performed an exhaustive swimming exercise test under 5% BW loading, their intrinsic endurance was ranked according to exhaustive swimming performance (Figure 1). The distribution pattern of exhaustive swimming exercise was similar to that reported by Koch et al. (1998). Koch and Britton (2001) demonstrated that of 96 male mice, the 13 lowest and the 13 highest performers became exhausted after an average of 13.0 ± 0.45 and 31.1 ± 0.81 min of running, respectively; thus, the exercise capacity of HEC mice was 2.4-fold higher than that of LEC mice. By contrast, in this study, of the 100 male mice, the 15 lowest and the 15 highest performers became exhausted after an average of 4.35 ± 0.23 and 172.40 ± 23.79 min of swimming, respectively; thus, the exercise capacity of HEC mice was 39.6-fold higher than that of LEC mice (Figure 2A). As illustrated in Figure 2B, the results of the grip strength test were similar to those of the exhaustive swimming exercise test. The grip strength of HEC mice (139.2 ± 5.1 g) was higher than that of the LEC (124.9 ± 3.2 g) and MEC (129.5 ± 3.2 g) mice (*p* < 0.05), indicating that the physical performance of HEC mice was significantly higher than that of MEC and LEC mice. Koch and Britton (2001) also detailed that selection for running capacity produced changes in BW and that the BW did not differ between HEC and LEC mice in the first generation; however, after breeding, the LEC- and HEC-line males exhibited significant differences in the fourth and fifth generations, respectively. In this study, BW and the growth curve did not differ significantly among LEC, MEC, and HEC mice (Table 1), possibly because these mice were not artificially selected for intrinsic aerobic endurance capacity. Swallow et al. (2010) demonstrated that HCR rats consumed and digested more food than LCR rats. Furthermore, HCR rats exhibited hypertrophy of the heart and kidneys and decreased length of the long intestine; thus, the oxygen utilization of these rats increased because of increased physical activity, resulting in increased food consumption and higher hypertrophy of key organs for O_2_ transport compared with LCR rats. Moreover, Novak et al. (2009) demonstrated that HCR rats exhibited increased physical activity, potentially leading to a degree of self-training and resulting in increased energy expenditure, reduced body fat, and other lean characteristics. By contrast, in the present study, no significant differences in the relative weights of organs (liver, kidney, heart, lung, EFP, or BAT) were evident between HEC and LEC mice, probably because our mice were not artificially selected generations. However, the muscle weight of HEC mice was significantly higher than that of LEC mice, consistent with the results of the physical activity tests (exhaustive swimming exercise and grip strength test); this may explain why HEC mice exhibited more neuromuscular coordination and muscular power improvement than LEC mice.

Exercise fatigue mechanisms can be generally divided into peripheral and central fatigue. Some common blood-based biological indicators can assess the physiological state of peripheral fatigue, including exercise fatigue indicators such as lactate, ammonia, BUN, and glucose and exercise-induced injury indicators (such as AST, ALT, CK, and LDH) (Huang et al., 2015). Although altering the peripheral system at the skeletal muscle level may facilitate the maintenance of endurance capacity, altering neural circuits regulating fatigue may be effective. Foley et al. (2006) demonstrated that increased brain 5-hydroxytryptamine (5-HT) release accelerates exercise fatigue, whereas increased brain dopamine (DA) release can delay it. These results suggest that individual differences in endurance capacity may be attributed to factors influencing the activities of 5-HT and DA systems. HCR rats exhibited higher 5-HT1B autoreceptor mRNA levels in the raphe nucleus and higher DR-D2 autoreceptor mRNA levels in the midbrain and striatum than LCR rats, indicating that central serotonergic and dopaminergic systems may be involved in the delay of exercise fatigue in HCR rats (Foley et al., 2006). In the present study, the levels of the fatigue indicators lactate, ammonia, CK, and AST were significantly lower in HEC mice than in LEC mice, possibly because of the positive effect of exercise on relevant metabolic mechanisms and circulatory systems; thus, the exercise performance of HEC mice was significantly higher than that of LEC mice. Ritchie et al. (2013) demonstrated that low intrinsic aerobic capacity is associated with increased risk factors for cardiovascular disease because of impaired glucose tolerance and elevated plasma insulin. In the present study, glucose levels in LEC mice were higher than those in MEC and HEC mice. Stephenson et al. (2012) demonstrated that the fasting serum insulin concentration in LCR rats was 62% higher than that in HCR rats; therefore, HCR rats had higher glucose tolerance. Ritchie et al. (2013) also determined that LCR rats had impaired glucose tolerance and upregulated gene expression of the glucose transporter GLUT4 compared with HCR rats. Previous studies used the HCR/LCR rat model, which is selected over many generations. However, in the current study, the mice were characterized by their aerobic exercise ability (without artificial population selection as F_0_ generation). Additionally, intrinsic LEC is associated with an increase in insulin-related metabolic syndrome, regardless of the intrinsic exercise capacity selected at many generations or at an early point in time (Stephenson et al., 2012; Ritchie et al., 2013).

The gut microbiota is associated with diseases such as obesity, aging, diabetes, allergies, cardiovascular disease, and cancer. Burcelin et al. (2011) discovered that the gut microbiota is involved in host metabolism, including adipose tissue functioning, liver fat storage, skeletal muscle energy metabolism, hepatic lipid metabolism, hepatic steatosis, atherosclerosis and cardiovascular diseases, tissue lipid composition in the retina and lens, periodontitis, behavior and motor activity, and enteroendocrine metabolism. Appukutty et al. (2015) demonstrated that the intervention combining *Lactobacillus plantarum* LAB12 intake and moderate exercise enhances functions and inhibits TNF-α production. Evans et al. (2014) reported that dietary intake and exercise intervention can alter the gut microbiota and produce physiological changes, such as weight control and high glucose tolerance. Hsu et al. (2015) determined that mice free of a specific pathogen exhibit improved exercise compared with completely pathogen-free mice, indicating that the composition of the gut microbiota is essential for exercise performance. In this study, we assessed whether the levels of intrinsic exercise capacity affect gut microbiota. In terms of genera, the numbers of *Anaeroplasma*, *Anaerovorax*, *Erysipelotrichaceae*, *Gemmiger*, and *Parasutterella* were significantly lower in HEC mice than in LEC mice, whereas the numbers of *Butyricicoccus* were significantly higher in LEC mice. Furthermore, *Butyricicoccus pullicaecorum* adapts to the gastrointestinal environment, is potentially probiotic (Geirnaert et al., 2014), and can improve inflammatory bowel disease (Steppe et al., 2014). Overall, our results indicate that HEC mice have a greater abundance and diversity of gut microbiota than LEC mice, which suggests that gut microbiota may be highly correlated with exercise capacity. In addition, the ratio of Firmicutes/Bacteroidetes was significantly higher in HEC mice than in LEC mice (Supplementary Figure 3). Exercise in normal rats was associated with higher numbers of Bacteroidetes and lower Firmicutes in fecal matter (Queipo-Ortuño et al., 2013; Evans et al., 2014). By contrast, the opposite was observed in exercised mice exposed to polychlorinated biphenyls (Choi et al., 2013) and also to type 2 diabetic (db/db) and control (db/^+^) mice (Lambert et al., 2015), similar to our own observations in HEC and LEC mice.

miRNAs are a class of highly abundant non-coding RNA molecules that can cause posttranscriptional gene repression; these are involved in various biological processes (Chaveles et al., 2012). In the present study, miR-383, miR-107, miR-30b, miR-669m, miR-191, miR-218, and miR-224 were significantly upregulated in HEC mice (Table 4). miR-383 regulates RBMS1 and inhibits downstream c-MYC expression, and along with miR-320, it controls E2F1 and SF1 genes to affect a steroid hormone. Therefore, miR-383 has therapeutic potential for hormone-related disorders (Yin et al., 2014). Van Caenegem et al. (2015) determined that an increase in testosterone levels can significantly increase muscle, power, and bone growth. miR-107 modulates the expression of the caveolin-1 gene, which encodes an upstream regulatory protein of the insulin receptor, thus improving insulin sensitivity. In this study, the glucose levels in LEC mice were higher than those in MEC and HEC mice; this may be related to the significant upregulation of miR-107 expression in HEC mice. In addition, miR-191 and miR-224 negatively regulate lipogenesis and inhibit lipid synthesis by modulating acyl-CoA synthetase activity (Ji et al., 2014). The proportion of muscle fat to visceral fat in HEC rats was lower than that in LEC rats (Spargo et al., 2007). In the present study, the EFP and BAT did not differ significantly among LEC, MEC, and HEC mice. However, future studies should investigate the influence of miR-191 and miR-224 on lipid regulation in artificially selected generations. Druz et al. (2012) determined that the oxidative stress induced by glucose deprivation caused reactive oxygen species accumulation and reduced glutathione depletion, which together inhibited histone deacetylase (HDAC) activity, reduced protein levels of HDAC2, and increased acetylation in miR-466 and miR-669 promoter regions, causing the activation of miR-466 and miR-669 to regulate physiological functions. The oxidative stress induced by glucose deprivation is similar to the exercise-induced oxidative stress observed in this study. This may explain why the levels of exercise-fatigue-related indicators, such as lactate and ammonia, were higher in LEC mice than in HEC mice. Chaveles et al. (2012) determined that the expression levels of miR-218 and miR-30 in liver tissue were higher after liver regeneration induced by two-thirds partial hepatectomy, suggesting that miR-218 and miR-30 may be crucial for liver regeneration. In this study, the mechanism of recovery from exercise-induced muscle damage was similar to that of liver regeneration. Therefore, the levels of injury-related biomarkers, such as CK, significantly decreased in HEC mice compared with those in LEC mice. These results indicate that these miRNAs related to intrinsic HEC play a major role in the adaptation and regulation of physiological functions.

The soleus muscle proteins exhibiting changes in regulation because of intrinsic exercise capacity were related to six major pathways between HEC and LEC mice, as determined by IPA, with a threshold *p* of <0.05. The six major biofunctions in the intrinsic exercise capacity-regulated soleus muscle proteins were muscle contraction, cardiovascular disease and injury, skeletal and muscle function, carbohydrate metabolism, muscle cell morphology, and organ morphology. The upregulated proteins associated with muscle contraction in HEC mice were ACTA1, ACTC1, Actin3, MYBPC1, MYBPC2, RYR1, and TNNT3. The muscle contraction was stronger in HEC mice than in LEC mice. Therefore, muscle weight, exhaustive swimming exercise performance, and grip strength were significantly higher in HEC mice than in LEC mice. Furthermore, low aerobic capacity may be associated with high risks of various diseases (Wisløff et al., 2005). Addressing associations and mechanisms concerning the progression of disease development using the respective indexes would be worthwhile. In addition, the results of different protein analysis on the functional network showed that the intrinsic exercise capacity is highly related to organ morphology and skeletomuscular development and function (Supplementary Figure 4). Thus, muscle function and carbohydrate metabolism may play an essential role in enhancing exercise performance.

In previous study with early obesity and non-alcoholic fatty liver disease (NAFLD) rat model, the exercise intervention could not only ameliorate HFD-induced body metabolic syndrome and hepatic steatosis, as a result of its lipid metabolism modulatory capacity and the exercise training also increased *Parabacteroides*, *Bacteroides*, and *Flavobacterium* genera, correlating with a beneficial metabolomic profile (Carbajo-Pescador et al., 2019). In our study, we also observed the *Bacteroides* genus is significantly higher proportions in HEC group than LEC group (Supplementary Figure 3) but the body composition and metabolic syndrome were not significant difference possible due to several reasons including, F_0_ generation, diet, and exercise intervention. There was another study to compare the diet and exercise on metabolic function and gut microbiota in obesity rat. The exercise intervention for obesity rat could reduce the inflammatory markers in all adipose tissue, and improve the BAT mitochondrial function, insulin resistance, and the relative abundance of Streptococcaceae (Welly et al., 2016). The GOTT test also showed that the HEC group exhibited the better glucose tolerance effects (Figure 5), which was also consistent with upregulation of miR-107 expression in HEC mice for insulin sensitivity (Table 4). In addition, the HEC also showed the higher expression levels in carbohydrate metabolism proteins (Supplementary Figure 3) related to aerobic metabolism. However, the diet and exercise training didn’t intervene HEC and LEC groups in current study, and the effects of gut microbiota and functional proteins with intrinsic exercise capacity need to be validated by further experimental designs by diet and training conditions.

One limitation of the study was sex of the mice used, as we used only male mice, and sex hormones can influence physiology and physiological adaptations. However, our experimental design focused on possible regulatory factors through a multiomics approach. Therefore, sex differences can be investigated in the future. In addition, ICR mice were subjected to an exhaustive swimming test and were ranked based on the exhaustive swimming time to distinguish intrinsically high- and low-capacity groups in the study. Previous studies have used the exhaustive swimming time method to assess the exercise capacity (Kan et al., 2018; Tung et al., 2019). However, mice are afraid of water, and the exhaustive swimming test causes them high mental stress. The blood biochemical parameters assessed in the present study (lactate, ammonia, BUN, glucose, LDH, CK, AST, and ALT) are not related to mental stress. Nevertheless, future studies should validate the changes in mental stress-related blood biochemical data, including hormones.

## Conclusion

In this study, exhaustive swimming test was used to determine low and high capacities in mice. In contrast with LEC mice, glucose tolerance and the number of *Butyricicoccus* were significantly higher in HEC mice, and levels of exercise-induced peripheral fatigue and injury-related biomarkers, including lactic acid, ammonia, CK, and AST, were significantly lower in HEC mice. In addition, differences between the exercise performance of HEC and LEC mice may be attributed to the expression of miR-383, miR-107, miR-30b, miR-669m, miR-191, miR-218, and miR-224, which are strongly associated with physiological and metabolic functions. Moreover, the functional protein profile indicated the effect of key protein levels related to intrinsic exercise capacity on muscle function and carbohydrate metabolism; these proteins may play a major role in the adaptive mechanism of exercise physiology. The mechanisms underlying the influence of these critical factors on disease development warrants further study.

## Data Availability

All datasets for this study are included in the manuscript and/or the Supplementary Files.

## Ethics Statement

This study was conducted in accordance with the principles of the Basel Declaration and the recommendations of the Institutional Animal Care and Use Committee (IACUC) of the NTSU. The protocol (IACUC-10103) was approved by the IACUC of the NTSU.

## Author Contributions

W-CH and C-CH designed the experiments. W-CH, Y-TT, Y-JH, and C-CL conducted the laboratory experiments. C-CH contributed the reagents, materials, and analysis platforms. S-TH analyzed the metagenome result. W-CH, Y-TT, Y-JH, and C-CH analyzed and illustrated the data. Y-TT, W-CH, and C-CH interpreted the results, prepared the figures, and wrote and revised the manuscript.

## Conflict of Interest Statement

The authors declare that the research was conducted in the absence of any commercial or financial relationships that could be construed as a potential conflict of interest.
