# Corrigendum: Physiological and Biochemical Effects of Intrinsically High and Low Exercise Capacities Through Multiomics Approaches

**DOI:** 10.3389/fphys.2019.01327

**Published:** 2019-10-17

**Authors:** Yu-Tang Tung, Yi-Ju Hsu, Chen-Chung Liao, Shang-Tse Ho, Chi-Chang Huang, Wen-Ching Huang

**Affiliations:** ^1^Graduate Institute of Metabolism and Obesity Sciences, Taipei Medical University, Taipei, Taiwan; ^2^Nutrition Research Center, Taipei Medical University Hospital, Taipei, Taiwan; ^3^Cell Physiology and Molecular Image Research Center, Wan Fang Hospital, Taipei Medical University, Taipei, Taiwan; ^4^Graduate Institute of Sports Science, National Taiwan Sport University, Taoyuan City, Taiwan; ^5^Proteomics Research Center, National Yang-Ming University, Taipei, Taiwan; ^6^Agricultural Biotechnology Research Center, Academia Sinica, Taipei, Taiwan; ^7^Department of Exercise and Health Science, National Taipei University of Nursing and Health Sciences, Taipei, Taiwan

**Keywords:** intrinsic exercise capacity, physical activities, gut microbiota, transcriptome, proteome

In the original article, there was a mistake in Figure 5 as published. The incorrect Figure 5 was erroneously uploaded at submission. Furthermore, all data should be represented as mean with SD, however, the original Figure 5 shows mean with SEM. Lastly, the statistical results in the original Figure 5 are correct, however, the way in which it is displayed is incorrect. The statistical letters should start from a, b, and c according to the mean average. The corrected Figure 5 appears below.

**Figure 5:**
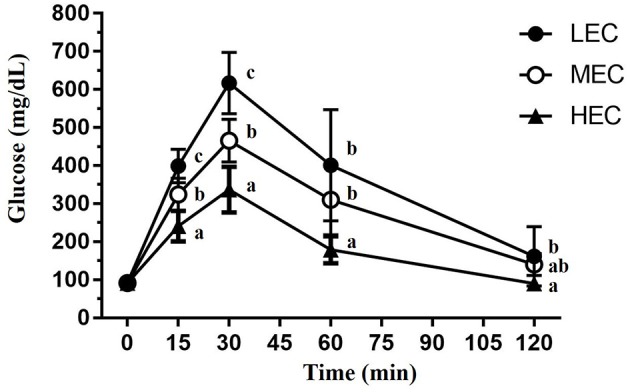
The oral glucose tolerance test was performed in mice with various exercise capacities at the same glucose dosage (2 g/kg) after 14 h of fasting. The indicated sampling times (0, 15, 30, 60, and 120 min) were plotted as the tolerance curve. Data are presented as mean ± standard deviation for 15 mice in each group. Different letters (a,b,c) indicate a significant difference at *p* < 0.05 using one-way ANOVA.

The authors apologize for this error and state that this does not change the scientific conclusions of the article in any way. The original article has been updated.

