# Supplementary material for: Physiological and Biochemical Effects of Intrinsically High and Low Exercise Capacities Through Multiomics Approaches
Source: Front Physiol. 2019 Sep 18;10:1201. doi: 10.3389/fphys.2019.01201 (PMC6759823; doi:10.3389/fphys.2019.01201)

**Supplementary Figure 1.** Heat map of miRNA differential expression among low exercise capacity (LEC), medium exercise capacity (MEC), and high exercise capacity (HEC) mice.


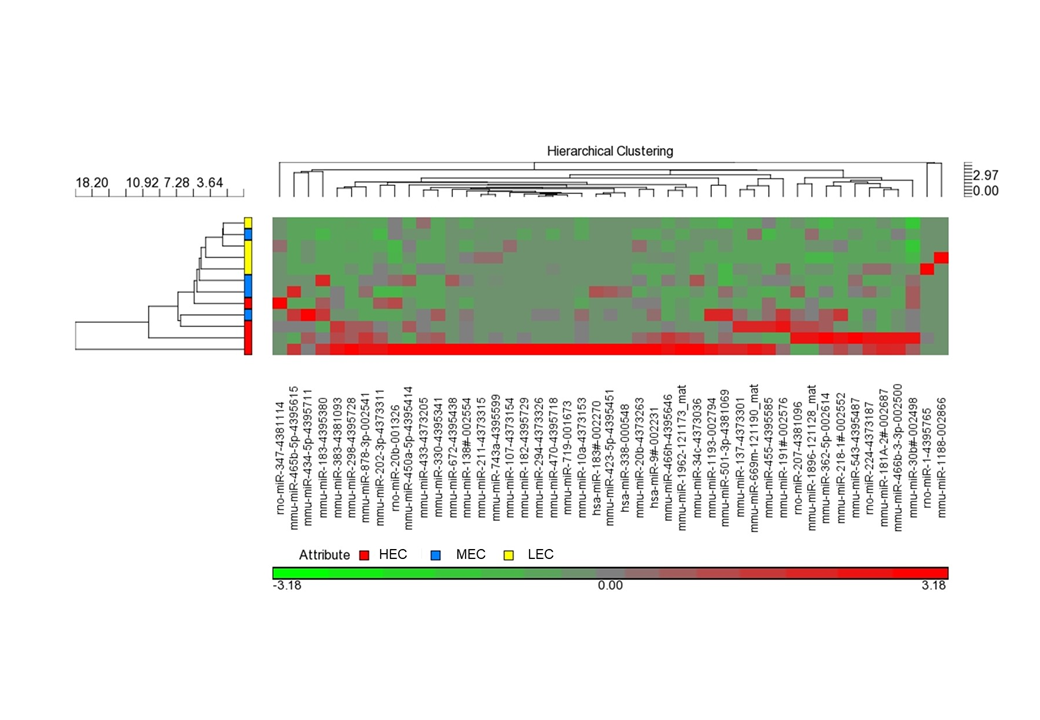


**Supplementary Figure 2.** Effect of exercise capacity on the significant expression of proteins. The six pathways with the greatest low exercise capacity–high exercise capacity differentials are included in the figure, as ranked by the significance between low exercise capacity and high exercise capacity mice. The vertical line indicates a threshold of P < 0.05.


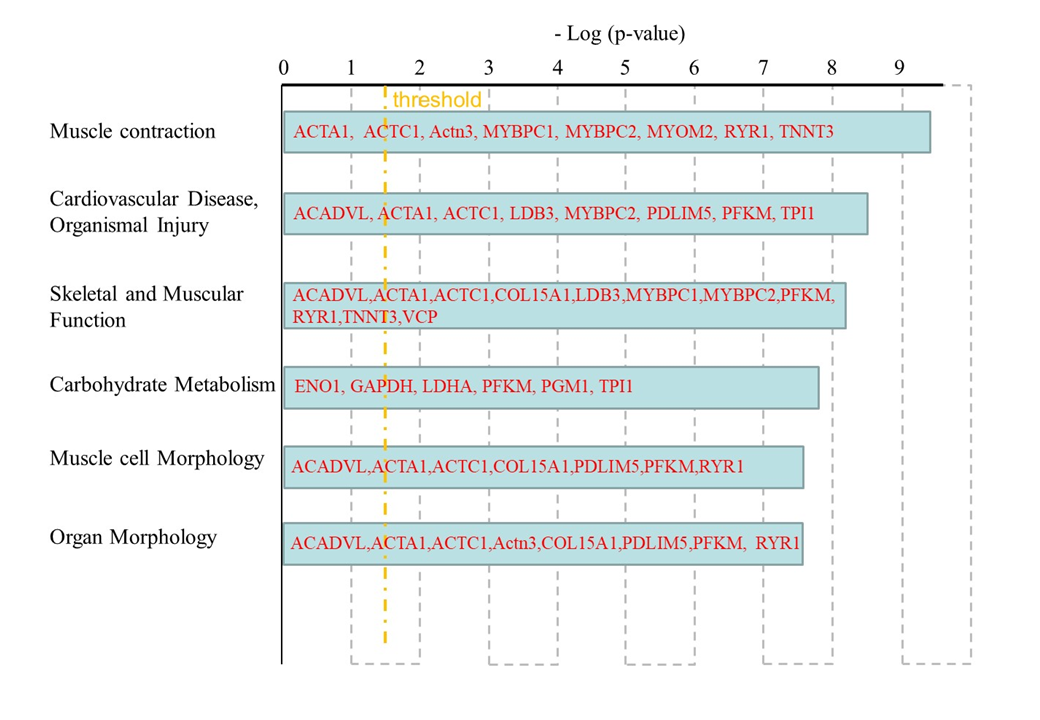


**Supplementary Figure 3.** The distribution of fecal microbiota was illustrated as pie charts in different classification to compare the difference between low exercise capacity (LEC) and high exercise capacity (HEC).


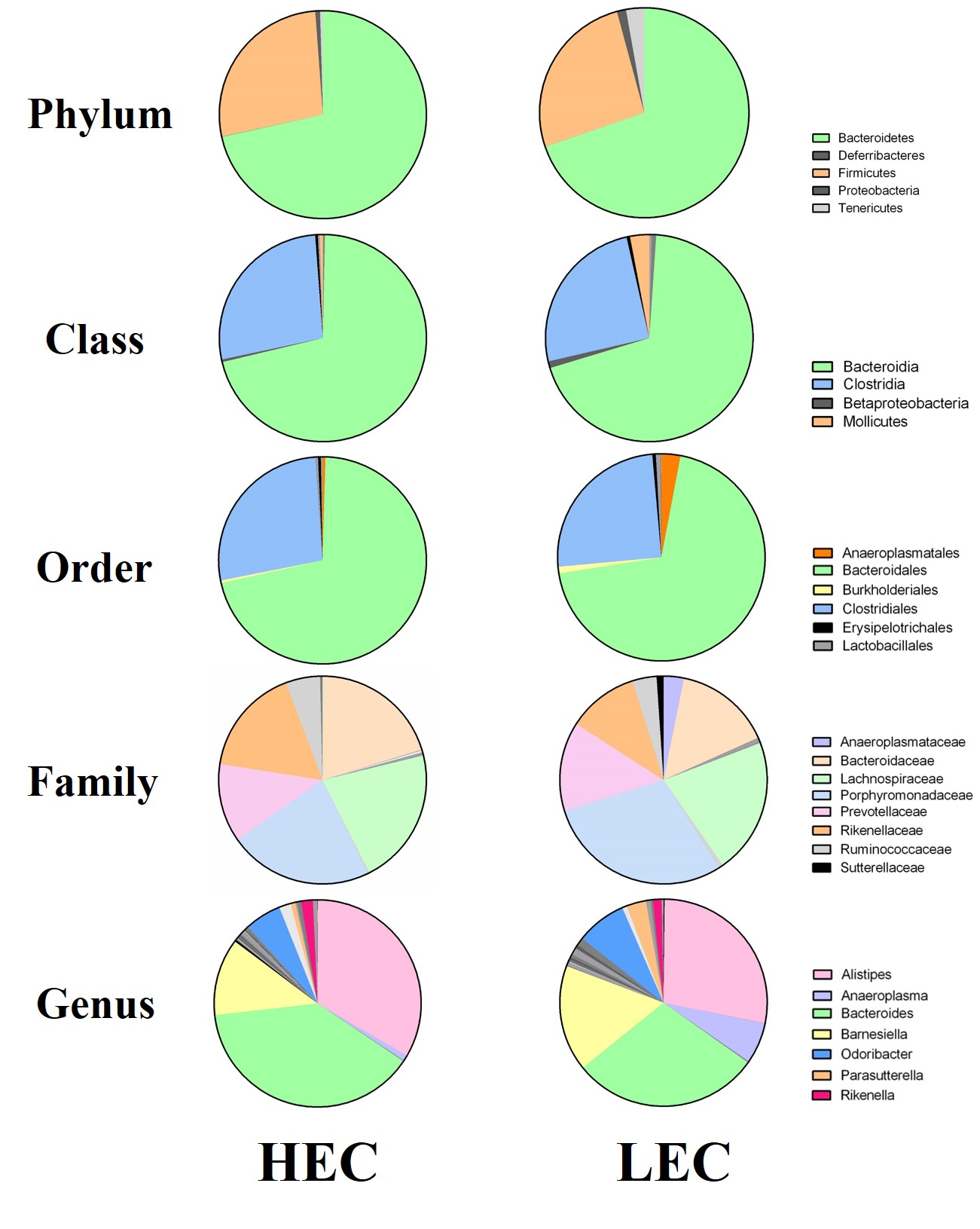


**Supplementary Figure 4.** Differential protein expression network relationships between low exercise capacity (LEC) and high exercise capacity (HEC) mice.


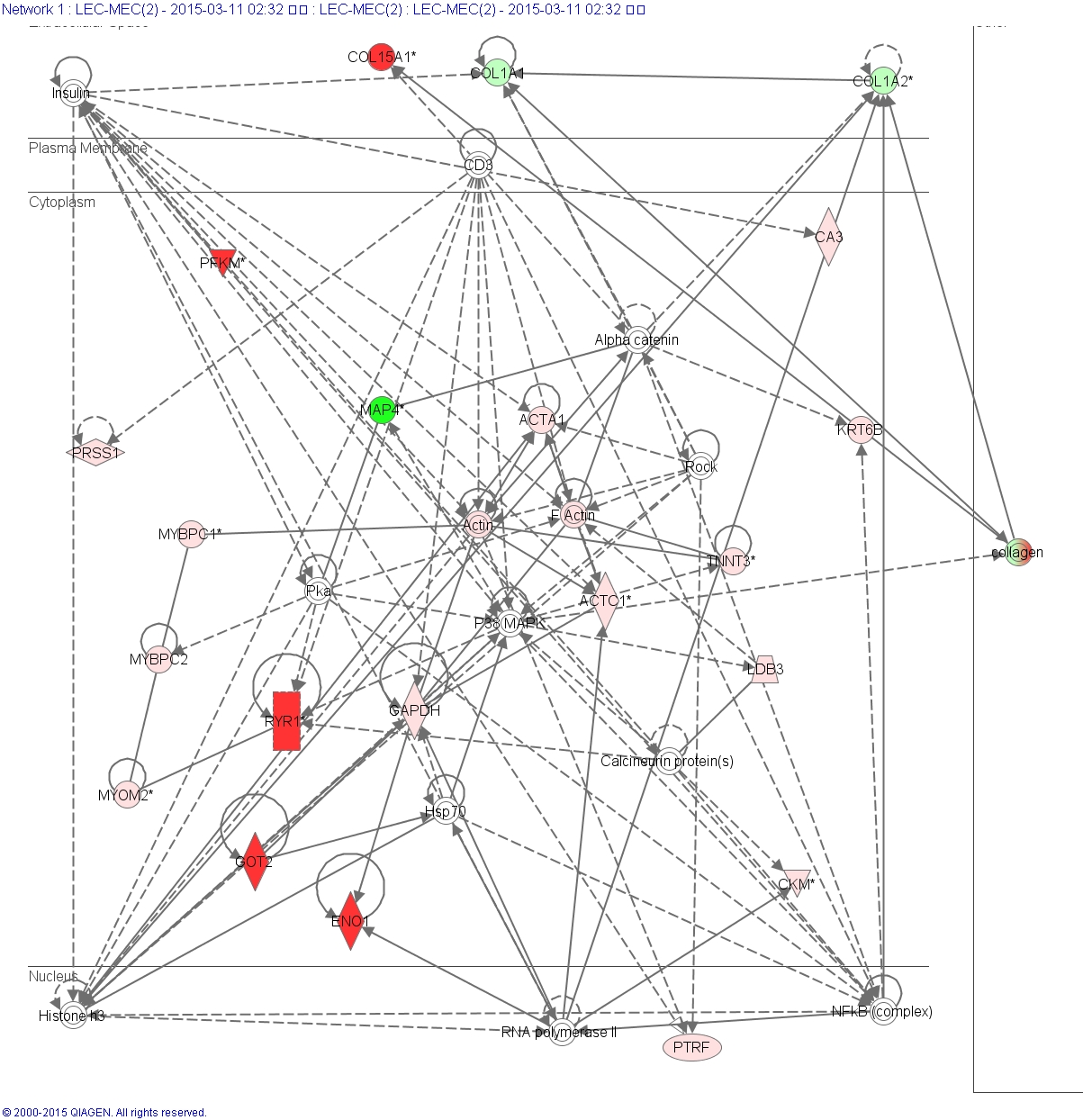

Supplement: Supplementary file 1 [file Data_Sheet_1.docx]
